# Neural responses to others’ pain vary with psychopathic traits in healthy adult males

**DOI:** 10.3758/s13415-015-0346-7

**Published:** 2015-03-17

**Authors:** Ana Seara-Cardoso, Essi Viding, Rachael A. Lickley, Catherine L. Sebastian

**Affiliations:** 1 Division of Psychology and Language Sciences, University College London, 26 Bedford Way, London, WC1H 0AP UK; 2 CIPsi, School of Psychology, University of Minho, Minho, Portugal; 3 Institute of Cognitive Neuroscience, University College London, London, UK; 4 Department of Psychology, Royal Holloway, University of London, London, UK

**Keywords:** Empathy, Psychopathic personality, fMRI, Anterior insula, Midcingulate gyrus, Inferior frontal gyrus

## Abstract

Disrupted empathic processing is a core feature of psychopathy. Neuroimaging data have suggested that individuals with high levels of psychopathic traits show atypical responses to others’ pain in a network of brain regions typically recruited during empathic processing (anterior insula, inferior frontal gyrus, and mid- and anterior cingulate cortex). Here, we investigated whether neural responses to others’ pain vary with psychopathic traits within the general population in a similar manner to that found in individuals at the extreme end of the continuum. As predicted, variation in psychopathic traits was associated with variation in neural responses to others’ pain in the network of brain regions typically engaged during empathic processing. Consistent with previous research, our findings indicated the presence of suppressor effects in the association of levels of the affective-interpersonal and lifestyle-antisocial dimensions of psychopathy with neural responses to others’ pain. That is, after controlling for the influence of the other dimension, higher affective-interpersonal psychopathic traits were associated with reduced neural responses to others’ pain, whilst higher lifestyle-antisocial psychopathic traits were associated with increased neural responses to others’ pain. Our findings provide further evidence that atypical function in this network might represent neural markers of disrupted emotional and empathic processing; that the two dimensions of psychopathy might tap into distinct underlying vulnerabilities; and, most importantly, that the relationships observed at the extreme end of the psychopathy spectrum apply to the nonclinical distribution of these traits, providing further evidence for continuities in the mechanisms underlying psychopathic traits across the general population.

**Electronic supplementary material:**

The online version of this article (doi:10.3758/s13415-015-0346-7) contains supplementary material, which is available to authorized users.

*Empathy* is a multidimensional phenomenon that involves the capacity to resonate with and understand the affective states of others (e.g., Singer & Lamm, 2009). It likely comprises both cognitive and affective components. One affective component, termed “affective resonance,” involves experiencing an affective state elicited by the observation or imagination of another person’s affective state. This experience, particularly in response to others’ distress, is thought to play a crucial role in appropriate social interaction. For example, it is thought that experiencing an affective response to others’ distress can elicit prosocial behavior (Nichols, 2001), whilst the absence of such a response can lead to a failure to inhibit aggression toward others (Blair, 2013; Blair, Mitchell, & Blair, 2005). Ultimately, a blunted empathic response system may lead to the development of inappropriate moral behavior (Blair et al., 2005).

Neuroimaging studies have utilized a wide range of different experimental tasks and stimuli (e.g., watching another person in painful situations, seeing a loved one about to receive an electric shock, or viewing another person expressing disgust) to probe the neural bases of empathy (see Fan, Duncan, de Greck, & Northoff, 2011, for a comprehensive review). Meta-analyses of these studies (Fan et al., 2011; Lamm, Decety, & Singer, 2011) have indicated that the observation of others’ experiences of distress, and more specifically of others’ experiences of pain, consistently elicits robust activation in anterior insula (AI), inferior frontal gyrus (IFG), and a region spanning the border between midcingulate cortex (midCC) and anterior cingulate cortex (ACC).

Callous and unempathic behavior is the hallmark of psychopathy, a personality disorder characterized by a constellation of traits including affective-interpersonal traits, such as lack of consideration for others’ feelings and a tendency to manipulate others, and lifestyle-antisocial behavior characteristics, such as impulsiveness and persistent antisocial behavior (Hare, 1993; Hare & Neumann, 2008). It has been proposed that the absence of a robust spontaneous empathic response to others’ distress explains why individuals with psychopathy find it easier to commit acts of antisocial behavior toward others (Blair, 2013; Blair et al., 2005). Indeed, both behavioral and neuroimaging data are consistent with the notion that these individuals do not find other people’s distress as salient as their peers do (see Blair, 2013, for a recent review). For example, individuals with extreme levels of psychopathic traits present a profile of blunted emotional reactivity to aversive stimuli including pictures of mutilated bodies and physical assault (Levenston, Patrick, Bradley, & Lang, 2000; Patrick, Bradley, & Lang, 1993), impaired recognition of distress cues in others (Blair, Colledge, Murray, & Mitchell, 2001; Blair et al., 2004; Blair et al., 2002), and atypical neural responses to stimuli depicting others experiencing pain in the network of brain regions typically recruited during empathic processing (i.e., AI, IFG, and midCC/dACC; Decety, Chen, Harenski, & Kiehl, 2013; Decety, Skelly, & Kiehl, 2013; Lockwood et al., 2013; Marsh et al., 2013; Meffert, Gazzola, Den Boer, Bartels, & Keysers, 2013).

The study of psychopathy in the general population has been the subject of considerable attention recently. There seems to be increasing interest in the subject, whether this relates to the influence of these traits in the workplace or the prevalence of high levels of these traits in people who hold key positions in society, such as in politics or banking. Research has now shown that the structure of psychopathic personality is dimensional rather than categorical; that is, psychopathic traits are normally distributed in the general population, and individuals with a diagnosis of psychopathy represent an extreme end of that distribution (see Hare & Neumann, 2008, for a review). Findings from studies inspecting the behavioral and neurophysiological correlates of psychopathic traits in the general population seem to mirror those observed in clinical/forensic samples and suggest that continuities in the mechanisms underlying psychopathy may exist (see Koenigs, Baskin-Sommers, Zeier, & Newman, 2011; Seara-Cardoso & Viding, 2014, for recent reviews).

With regard to empathic processing, evidence suggests that high levels of psychopathic traits in the general population are associated with reduced emotional reactivity to aversive stimuli (e.g., Benning, Patrick, & Iacono, 2005; Justus & Finn, 2007), as well as with weaker self-reported affective responses to others’ emotional faces (Ali, Amorim, & Chamorro-Premuzic, 2009; Seara-Cardoso, Dolberg, Neumann, Roiser, & Viding, 2013; Seara-Cardoso, Neumann, Roiser, McCrory, & Viding, 2012). At the neural level, evidence suggests that in the general population psychopathic traits are associated with atypical responses in brain regions including IFG, ventromedial prefrontal cortex and amygdala when processing emotional facial expressions (Carré, Hyde, Neumann, Viding, & Hariri, 2013; Gordon, Baird, & End, 2004; Hyde, Byrd, Votruba-Drzal, Hariri, & Manuck, 2014), and when punishing others with electric shocks (Molenberghs et al., 2014). And when rating one’s own affective response to others’ emotional faces (Seara-Cardoso, Sebastian, Viding, & Roiser, under review). These findings suggest that links between psychopathy and poor empathic responding extend throughout the continuum of psychopathic traits at both the behavioral and neural levels.

There is also clear evidence that youth and adults with extreme levels of psychopathic traits show atypical neural responses to others’ pain when compared with healthy controls (Decety et al., 2013a, b; Lockwood et al., 2013; Marsh et al., 2013; Meffert et al., 2013). However, there is little evidence to suggest whether neural responses to others experiencing pain similarly vary continuously with psychopathic traits in the general adult population. Here, we employed the imaging paradigm and analysis strategy previously described by Lockwood et al. to study whether individual variability in neural responses to others’ pain is associated with psychopathic traits in the general population. Lockwood et al. measured fMRI responses to pictures of others’ hands and feet either in pain or in no pain (control condition) in a large sample of children with conduct problems and typically developing controls. As predicted, the children with conduct problems exhibited significantly reduced neural responses in regions previously associated with empathic processing—namely AI, IFG, and ACC—in comparison to the typically developing control group. However, considerable heterogeneity of neural responses was seen within the conduct problems group. When callous traits (similar to adult affective-interpersonal psychopathic traits) and conduct disorder symptoms (similar to adult lifestyle-antisocial behavior characteristics) were analyzed together as continuous independent variables in regression analyses, neural responses to others’ pain were negatively associated with callous traits (in AI and ACC), but positively associated with conduct disorder symptoms (in ACC). These relationships only became apparent when the unique contribution of each of these variables was inspected, controlling for the other.

This pattern of opposing relationships between the two dimensions of psychopathic traits and measures of affective processing, with relationships only emerging after shared variance is controlled for, is consistent with research to date that has suggested that these two dimensions exert suppressor effects on each other (e.g., Blonigen et al., 2010; Hicks & Patrick, 2006; Vanman, Mejia, Dawson, Schell, & Raine, 2003). Suppression, in this case cooperative suppression, occurs when two correlated variables (as is the case for the two dimensions of psychopathic traits) present opposing relationships with a given criterion variable, such that the inclusion of both concurrently in a regression model increases the association of each with the criterion variable (Watson et al., 2013). In other words, the association of each dimension of psychopathy is greater when the variance shared with the other dimension is accounted for, because variance shared with the other dimension does not present the same relationship with the criterion variable, and therefore suppresses the association (Blonigen et al., 2010). In psychopathy research, these suppressor effects seem to indicate that, although affective-interpersonal and lifestyle-antisocial features often co-occur and present shared components, unique aspects of each dimension (i.e., those not shared with the other dimension) are related to distinct types of atypical emotional and cognitive processing.

With respect to emotional processing, behavioral studies in both general and psychopathic samples have shown that, when holding the other dimension constant, the affective-interpersonal dimension (characterized by blunt affect and shallowness) is indeed associated with reduced reactivity to emotional stimuli, whilst the lifestyle-antisocial dimension (characterized by impulsivity and irresponsibility) is associated with increased reactivity to emotional stimuli (e.g., Blonigen et al., 2010; Hicks & Patrick, 2006; Seara-Cardoso et al., 2012; Uzieblo, Verschuere, van den Bussche, & Crombez, 2010; Vanman et al., 2003). This pattern of divergent associations between the two dimensions of psychopathy has also been found at the neural level in the amygdala (a region implicated in affective processing) in response to non-pain-related emotional stimuli in children with conduct problems (Lozier, Cardinale, VanMeter, & Marsh, 2014; Sebastian et al., 2012) and in typical adults (Carré et al., 2013; Hyde et al., 2014), as well as in AI and ACC during empathy processing in children with conduct problems, as we discussed above (Lockwood et al., 2013).

In sum, the extant evidence indicates that individuals with extreme levels of psychopathy present a pattern of reduced behavioral and neural response to others’ suffering, which may, in part, explain some of their characteristic inappropriate social interactions. However, we do not yet know whether neural processing of others’ pain relates to variability in psychopathic traits in those individuals who function in the community. We used the methodology described in Lockwood et al. (2013) to study whether neural responses to others’ pain vary with psychopathic traits within the general population, in a similar manner to responses among individuals with extreme levels of these traits. If the neurobiological correlates of psychopathy vary along a continuum in the general population, we would expect to find a pattern of neural responses in the brain regions typically recruited during empathic processing (i.e., AI, IFG, and mid/dACC), consistent with previous research based on individuals with extreme levels of psychopathic traits (Decety et al. 2013a, b; Lockwood et al., 2013; Marsh et al., 2013; Meffert et al., 2013). More specifically, consistent with the literature showing that the affective-interpersonal and lifestyle-antisocial dimensions of psychopathy may reflect distinct underlying vulnerabilities, we predicted that these two dimensions of psychopathy would exert suppressor effects on each other in relation to activity in these regions while viewing others’ pain.

## Method

### Participants

Fifty-three right-handed male participants from the community with no reported history of psychiatric illness were recruited for this study. Of these, six were excluded before preprocessing due to failure to complete the task (two participants), excessive response times (two participants), incidental findings (one participant), and corrupted fMRI data due to excessive movement (one participant). Analyses of the residuals from the multiple regression models inspecting the relationships between neural responses and psychopathic traits revealed one extreme outlier. This participant was excluded, leaving 46 participants in the analyses (mean age 27.93 years, range 19–40). According to the G*Power software (Faul, Erdfelder, Lang, & Buchner, 2007), a sample size between 38 (for one-tailed analyses) and 49 (for two-tailed analyses) is appropriate to detect an effect size of *R* ^2^ = .17, similar to the average effect size reported in Lockwood et al. (2013), at an alpha significance level of .05 with 80 % power. Thus, an appropriate sample size was recruited. All participants provided written informed consent according to the guidelines approved by UCL Division of Psychology and Language Sciences Ethics Committee, who provided ethical approval for this study.

### Experimental task

The stimuli were 192 digital photographs showing another person’s hand or foot in painful or nonpainful situations (taken from Gu et al., 2010). “Pain” and “no-pain” stimuli (96 pictures per condition) were matched on their physical properties and had been validated as eliciting empathy-related activations in a previous study (Gu et al., 2010). Stimuli were presented in 24 pain and no-pain blocks, each lasting 20 s and consisting of eight images, each displayed for 2,000 ms with a 500-ms interstimulus interval. Blocks were pseudorandomized, with the same block type never being presented more than twice in a row. A fixation cross was presented for 15 s every six blocks. To ensure attention, participants performed a hand/foot keypress judgment on every trial. Participants practiced outside the scanner with painful and nonpainful images not seen in the main experiment until ≥80 % accuracy was reached.

### Psychometric measures

Participants completed the Self-Report Psychopathy Scale, Short Form (SRP-SF; Paulhus, Neumann, & Hare, 2015), a 29-item scale designed to measure psychopathic attributes in non-institutionalized samples. The SRP-SF assesses psychopathic traits, organized in four facets—interpersonal, affective, lifestyle and antisocial—consistent with recent research on the Psychopathy Checklist–Revised (PCL-R; Hare, 2003). Higher scores on the SRP questionnaire reflect higher levels of psychopathic traits. Like the PCL-R, the four facets can be modeled in terms of the traditional two-factor dimensions: affective-interpersonal and lifestyle-antisocial. The SRP has been shown to have a clear latent structure and good construct validity (Mahmut, Menictas, Stevenson, & Homewood, 2011; Neumann & Pardini, 2014; Neumann, Schmitt, Carter, Embley, & Hare, 2012) and is strongly correlated with the PCL-R (Lilienfeld & Fowler, 2006; Paulhus et al., 2015). In the present sample, Cronbach’s alpha for the total SRP scale was .91, for the affective-interpersonal scale was .88 and for the lifestyle-antisocial scale was .84. Affective-interpersonal scores varied between 15 and 61 (*M* = 29.85, *SD* = 9.11), lifestyle-antisocial scores varied between 15 and 47 (*M* = 29.15, *SD* = 8.89), and the two scales presented a correlation coefficient of *r* = .66 (*p* < .001), thus presenting a distribution similar to previously reported distributions from larger samples of adults from the general population (Foulkes, Seara-Cardoso, Neumann, Rogers, & Viding, 2014; Foulkes, McCrory, Neumann, & Viding, 2014; Paulhus et al., 2015; Seara-Cardoso et al., 2012). Participants also completed the State–Trait Anxiety Inventory (STAI; Spielberg 1983), which comprises two subscales measuring trait and state anxiety. The Matrix Reasoning subscale of the Wechsler Abbreviated Scale of Intelligence (Wechsler, 1999) was administered to estimate general intellectual ability.

#### Magnetic resonance imaging acquisition

Images were acquired using a Siemens Avanto 1.5-T MRI scanner at the Birkbeck–UCL Centre for Neuroimaging with a 32-channel head coil. A total of 189 multislice T2*-weighted echo-planar images (EPIs) with blood oxygenation-level-dependent (BOLD) contrast were acquired in a single run of 9 min. The T2* EPI sequence was based on Weiskopf, Hutton, Josephs, and Deichmann (2006) and used the following acquisition parameters: 35 2-mm slices acquired in a descending trajectory with a 1-mm gap, echo time = 50 ms, repetition time = 2.975 s, slice tilt = –30°, flip angle = 90°, field of view = 192 mm, and matrix size = 64 × 64. A 5.5-min T1-weighted MPRAGE scan was acquired for coregistration, normalization, and overlay.

#### Image processing and analyses

The EPI data were analyzed using SPM8 (www.fil.ion.ucl.ac.uk/spm) in MATLAB. The first five and last two volumes were discarded. The data were realigned to the sixth volume, normalized to the Montreal Neurological Institute template (resampling to a voxel size of 2 × 2 × 2 mm), and smoothed with an 8-mm full-width-at-half-maximum Gaussian filter. The data were high-pass filtered at 128 s to remove low-frequency drifts, and the statistical model included an AR(1) autoregressive function to account for autocorrelations.

After preprocessing, a block analysis compared neural activity associated with the pain and no-pain conditions. Regressors included pain and no pain (blocks of 20 s duration) and fixation (15 s), modeled as boxcar functions convolved with a canonical hemodynamic response function. The six realignment parameters were also modeled as effects of no interest. At the first level, pain > no-pain and no-pain > pain contrasts were created. These contrast images were entered into second-level analyses, in which both SRP dimensions were entered either separately or simultaneously as covariates in multiple regression models. Relationships between the total SRP scores and BOLD response were also examined.

**Fig. 1:**
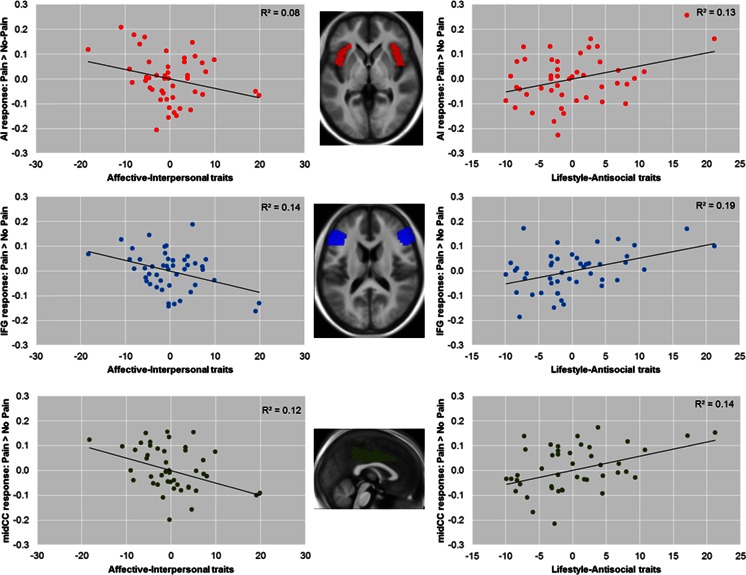
Partial regression plots showing opposing relationships between response to pain > no-pain in bilateral anterior insula (AI), inferior frontal gyrus (IFG), and midcingulate cortex (midCC), as well as unique variances associated with affective-interpersonal and lifestyle-antisocial psychopathic traits after the other dimension had been controlled for (a similar pattern was also seen in the anterior cingulate cortex [ACC], adjacent to midCC). (Left) Negative relationships between blood oxygenation-level-dependent (BOLD) response to pain > no-pain and affective-interpersonal traits after controlling for the effect of lifestyle-antisocial traits. (Right) Positive relationships between BOLD response to pain > no-pain and lifestyle-antisocial traits after controlling for the effect of affective-interpersonal traits. *R* ^2^ reflects the partial correlation coefficients of determination. The insets show horizontal and midsagittal sections of the bilateral AI (*z* = 0), IFG (*z* = 15), and midCC (*x* = 0) regions of interest, overlaid on an average T1 structural image from all participants

Whole-brain analyses for the pain > no-pain contrast are reported using a cluster-forming threshold of *p* < .001 (uncorrected, cluster size > 10), with cluster-level family-wise error (FWE) correction. Region-of-interest (ROI) analyses were conducted in four a priori ROIs (bilateral AI, IFG, ACC, and midCC). The first three were taken from Lockwood et al. (2013), and the midCC was added because it regularly features in meta-analyses of empathy for pain, with clusters bordering midCC and ACC (Fan et al., 2011; Lamm et al., 2011). ROI analyses were conducted as described by Lockwood et al. ROIs were anatomically defined using masks from the automated anatomical labeling atlas (Maldjian, Laurienti, Kraft, & Burdette, 2003), and the MarsBaR toolbox (http://marsbar.sourceforge.net) was used to calculate average contrast estimates across bilateral ROIs and to conduct *t* tests at a standard statistical threshold of *p* < .05 (Eisenberger et al., 2010; Masten et al., 2011). The contrast estimates extracted with MarsBaR were also used in IBM SPSS and MS Excel to conduct regression analyses and to generate the illustrative partial regression plots presented in Fig. 1 below.

## Results

### Behavioral data

Mean reaction times (RTs) and percentage error rates were calculated. Consistent with previous studies (Gu et al., 2010; Lockwood et al., 2013), RTs were significantly slower during pain than during no pain [*t*(45) = 5.76, *p* < .001; pain: *M* = 767.11, *SD* = 106.62; no pain: *M* = 738.10, *SD* = 104.52]. We found no significant differences in percentage error rates between the conditions (pain: *M* = 2.97, *SD* = 2.50; no pain: *M* = 2.72, *SD* = 2.36).

On the basis of previous studies showing that the unique variances associated with affective-interpersonal and lifestyle-antisocial traits present opposing associations with emotional reactivity (e.g., Carré et al., 2013; Hicks & Patrick, 2006; Lockwood et al., 2013; Lozier et al., 2014; Seara-Cardoso et al., 2012), we conducted two regression analyses in which both dimensions of psychopathy were entered as predictors of the difference in mean RTs for pain > no-pain and of the difference in percentage error rates for pain > no-pain, respectively. After controlling for levels of the other dimension, lifestyle-antisocial traits presented a significant positive association with the difference in percentage error rates (*t* = 2.253, *p* = .03), whilst affective-interpersonal traits presented an at-trend negative association (*t* = –1.72, *p* = .09). That is, when holding the other dimension constant, higher levels of affective-interpersonal traits were associated (at trend level) with fewer errors during the pain than during the no-pain condition, whilst higher levels of lifestyle-antisocial traits were associated with increased error rates in the pain than in the no-pain condition. There were no significant associations with the difference in RTs for the pain relative to the no-pain condition, and no significant bivariate associations between total SRP score or either dimension of psychopathic traits and the mean RT/percentage error rate differences between conditions (all *p*s > .30).

### Imaging results

The results from the whole-brain analyses for the main effect of pain > no-pain are displayed in Table 1 (see Supplementary Table S1 for no-pain > pain). Main effects were found in regions previously associated with empathy for pain, and these largely replicated those from previous studies using the same stimuli (Gu et al., 2010; Lockwood et al., 2013), including bilateral IFG extending to AI (*p* < .001, FWE-corrected at cluster level). ROI analyses for the main effect of pain > no-pain also revealed the predicted pattern of significant BOLD response in the bilateral AI [*t*(45) = 1.68, *p* = .05] and IFG [*t*(45) = 3.61, *p* < .001], but not in midCC [*t*(45) = 0.70, *p* = .24] and ACC [*t*(45) = –0.10, *p* = .34]. Additionally, entering the difference (pain > no-pain) in error rates and the difference in RTs between conditions as predictors of BOLD response in two SPM models also showed that BOLD response in all ROIs presented significant positive relationships with differences in error rates [AI, *t*(44) = 3.08, *p* < .01; IFG, *t*(44) = 2.08, *p* = .02; midCC, *t*(44) = 2.69, *p* < .01; and ACC, *t*(44) = 2.94, *p* < .01] and differences in RTs [AI, *t*(44) = 2.98, *p* < 0.01; midCC, *t*(44) = 1.69, *p* = 0.05; and ACC, *t*(44) = 1.97, *p* = 0.03] between the pain and no-pain conditions, with the exception of the IFG, whose association was at trend [*t*(44) = 1.05, *p* = .15].

**Table 1:** Whole-brain analyses showing main effects for the pain > no-pain blood oxygenation-level-dependent response

	Peak						Cluster	
Brain Regions	L/R	*x*	*y*	*z*	*t*	*Z*	Extent (*k*)	*p* (FWE)
Middle temporal gyrus	L	–44	–68	–2	11.75	>8	3540	<.001
Occipital gyrus	R	32	–84	1	10.83	7.56	3367	<.001
Supramarginal gyrus	L	–56	–30	32	10.10	7.26	1326	<.001
Supramarginal gyrus	R	66	–24	38	8.45	6.50	854	<.001
Precentral gyrus	L	–50	4	30	6.97	5.71	818	<.001
Cerebellum	R	16	–76	–50	6.21	5.25	191	.03
Inferior frontal gyrus	R	56	38	6	5.51	4.79	369	<.001
Insula	L	–38	–4	–10	4.79	4.48	81	.33
Precentral gyrus	R	50	6	30	4.48	4.27	388	<.001
Inferior frontal gyrus	L	–52	38	6	4.26	3.88	51	.60
Inferior frontal gyrus	L	–40	28	0	4.25	3.88	206	.03
Ext. insula	L	–32	28	4	4.17	3.82		
Postcentral gyrus	L	32	–34	42	4.25	3.88	29	.85
Amygdala	R	22	–4	–14	4.21	3.84	42	.70
Cerebellum	R	16	–74	–50	3.76	3.49	50	.61

Whole-brain analysis are reported at a threshold level of *p* < .001 (uncorrected, cluster size > 10 voxels). Spatial coordinates (*x*, *y*, *z*) are in Montreal Neurological Institute space. R = right, L = left, FWE = family-wise error corrected

On the basis of previous studies showing that the unique variances associated with affective-interpersonal and lifestyle-antisocial traits show opposing associations with emotional reactivity (e.g., Carré et al., 2013; Hicks & Patrick, 2006; Lockwood et al., 2013; Lozier et al., 2014; Seara-Cardoso et al., 2012), we entered both dimensions of psychopathy as predictors in a single multiple regression model at the second level in SPM and tested whether neural response in our ROIs was associated with each dimension individually, after controlling for the other (see Fig. 1). As predicted, ROI analyses for pain > no-pain revealed that, after controlling for lifestyle-antisocial traits, the unique variance associated with affective-interpersonal traits was negatively related to BOLD response in AI [*t*(43) = 1.87, *p* = .03], IFG [*t*(43) = 2.68, *p* < .01], and midCC [*t*(43) = 2.38, *p* = .01], and was at trend in ACC [*t*(43) = 1.24, *p* = .11]. That is, when holding levels of lifestyle-antisocial behavior constant, increased levels of affective-interpersonal traits were associated with a decrease in neural responses to others’ pain in these regions. After controlling for affective-interpersonal traits, the unique variance associated with lifestyle-antisocial traits was positively related to differential BOLD response in AI [*t*(43) = 2.51, *p* < .01], IFG [*t*(43) = 3.16, *p* < .01], midCC [*t*(43) = 2.64, *p* < .01], and ACC [*t*(43) = 1.92, *p* = .03]. That is, when holding levels of affective-interpersonal traits constant, increased levels of lifestyle-antisocial behavior traits were associated with an increase in neural responses to others’ pain in these regions.

To exclude the potential confounds of trait anxiety and cognitive ability, we included trait anxiety and estimated IQ as additional covariates in follow-up regression models. Including these variables did not change the pattern of results (all significant results remained at *p* < .05, with the exception of the association of lifestyle-antisocial traits and BOLD response in ACC, *p* = .12).

To test whether these opposing results were genuine suppressor effects, we inspected the bivariate associations between psychopathic dimensions and total score and differential BOLD response in three separate regression models. These analyses revealed weaker and largely nonsignificant bivariate associations between neural responses in our ROIs and affective-interpersonal traits [AI, *t*(44) = 0.27, *p* = .39; IFG, *t*(44) = 0.72, *p* = .24; midCC, *t*(44) = 0.80, *p* = .21; ACC, *t*(44) = 0.04, *p* = .97], lifestyle-antisocial traits [AI, *t*(44) = 1.66, *p* = .05; IFG, *t*(44) = 1.74, *p* = .44; midCC, *t*(44) = 1.36, *p* = .09; ACC, *t*(44) = 1.46, *p* = .15], and total psychopathy score [AI, *t*(44) = 0.72, *p* = .24; IFG, *t*(44) = 0.60, *p* = .55; midCC, *t*(44) = 0.27, *p* = .40; ACC, *t*(44) = 0.44, *p* = .22].

For completeness, due to previous research linking amygdala dysfunction to psychopathic traits, we inspected whether neural responses in amygdala varied as a function of psychopathic traits. No significant associations were found.

## Discussion

Neuroimaging studies have shown that the observation of other people experiencing distress, in particular pain, elicits robust activation in AI, IFG and midCC/ACC (Fan et al., 2011; Lamm et al., 2011). Consistent with the idea that individuals with extreme levels of psychopathy do not find other people’s distress as salient as their peers do (Blair et al., 2005), it has been reported that these individuals show atypical neural activity in these regions in response to others’ pain, when compared with healthy controls (Decety et al. 2013a, b; Lockwood et al., 2013; Marsh et al., 2013; Meffert et al., 2013). However, although affective dysfunction is considered to be a critical, defining feature of psychopathy (Blair et al., 2005), there has been little evidence as to whether empathic neural responses to others’ pain vary continuously with psychopathic traits in typical adults—that is, as to whether the pattern of relationships between psychopathic personality traits and neural response to others’ pain observed at the extreme end of the psychopathy distribution may also be observed in a nonclinical distribution of these traits in functioning members of the general population.

In line with predictions, we found that psychopathic traits were significantly associated with neural responses to stimuli depicting others’ experiencing pain in AI, IFG, and midCC/ACC. More specifically, we found suppressor effects between the two dimensions of psychopathy in terms of their relationships with neural responses to others’ pain in these regions. The unique variance associated with affective-interpersonal traits was negatively associated with neural responses to others’ pain in these regions, whilst at the same time the unique variance in lifestyle-antisocial traits was positively associated with neural responses.

It has been proposed that the AI, IFG, and midCC/ACC play separate but complementary roles in empathic processing. The AI is proposed to be critical for sensory integration (Critchley, Wiens, Rotshtein, Öhman, & Dolan, 2004), for the representation and integration of feeling states (Craig, 2009), and for effectively discriminating between emotionally salient and nonsalient information (Gu et al., 2012). The midCC/ACC, with extensive connections from the somatosensory cortices and to and from the insula, amygdala, ventral striatum, and periaqueductal gray, is thought to be a hub region in affective, cognitive, and motor control and, ultimately, to influence the motor centers responsible for expressing affect and executing goal-directed behavior (Bernhardt & Singer, 2012; Shackman et al., 2011). Whereas the AI is thought to serve as an input region of the system, translating sensations into subjective feelings and awareness, the cingulate may function as an output region, exerting volitional control (Gu et al., 2012). The IFG, on the other hand, is thought to play a role in emotional contagion and emotional recognition (Shamay-Tsoory, 2011), as well as in emotion regulation and pain suppression (Ochsner & Gross, 2005; Salomons, Johnstone, Backonja, Shackman, & Davidson, 2007; Wager, Davidson, Hughes, Lindquist, & Ochsner, 2008).

Psychopathic traits are characterized by lack of empathy, disregard for other people’s feelings, impulsiveness, and antisocial behavior. It would therefore be unsurprising to find that individuals with extreme levels of these traits presented atypical engagement of the regions outlined above when faced with others’ distress. We did not find significant bivariate associations between psychopathic traits and neural responses in these regions, which could have been due to sample size and lack of statistical power or to the limited range of scores in the extreme end of psychopathic traits in our sample, but associations became apparent once the two dimensions of psychopathic traits were inspected and their shared variance was controlled for. In line with previous research with clinical/forensic samples (e.g., Hicks & Patricks, 2006; Lockwood et al., 2013; Vanman et al., 2003), we found a cooperative suppression effect between the affective-interpersonal and lifestyle-antisocial dimensions of psychopathy and neural responses to others’ pain. This cooperative suppression effect occurs because the two dimensions of psychopathic traits are correlated with each other but present opposing relationships with neural responses to pain-related stimuli in these regions. The association of each dimension of psychopathy with neural responses becomes apparent when the shared variance is accounted for—that is, when the other dimension is held constant. The variance shared with the other dimension does not present the same relationship with the criterion variable, and therefore suppresses the association (Blonigen et al., 2010).

We also observed a pattern of cooperative suppression between the two dimensions of psychopathic traits and the difference in error rates between pain and no-pain conditions. More specifically, we found that when holding the other dimension constant, lifestyle-antisocial traits presented a positive association with the difference in error rates between conditions, whilst affective-interpersonal traits presented a negative association with the difference in error rates. That is, when holding affective-interpersonal traits constant, higher lifestyle-antisocial traits corresponded to a higher rate of errors made in the pain than in the no-pain condition, but when holding lifestyle-antisocial traits constant, higher affective-interpersonal traits corresponded to a reduced rate of errors made in the pain than in the no-pain condition (at trend levels). An increased error rate in the pain condition is thought to result from increased reactivity to the emotional content of the stimuli (i.e., depicting others’ pain in comparison to no pain) and consequent interference in task performance (hand/feet judgment) (Gu, Liu, Van Dam, Hof, & Fan, 2013).These results are in line with the notion that higher levels of affective-interpersonal traits are accompanied by less reactivity to stimuli depicting others’ pain (reflected by less “interference” from others’ pain and lower error rates), and higher levels of lifestyle-antisocial traits are accompanied by higher reactivity to the same stimuli (reflected by higher interference and higher error rates). Furthermore, we found significant positive associations between the difference in error rates and neural responses in all of the above-mentioned regions—that is, those participants who presented a greater difference in error rates in pain versus no-pain also presented higher neural responses in these regions. This corroborates the notion that individual differences in reactivity to others’ pain influence responses in the neural circuitry thought to be involved in empathy for pain.

We found that unique variances associated with the two dimensions of psychopathic traits, affective-interpersonal and lifestyle-antisocial, presented opposing associations with neural response to pain (relative to no pain) in AI, IFG, midCC, and ACC. After shared variance with lifestyle-antisocial traits was removed, affective-interpersonal traits (characterized by a lack of consideration for others’ well-being) presented negative associations with neural responses in AI, IFG, and midCC, which is consistent with the characteristic lack of arousal to others’ distress and the general blunted affect associated with this set of traits. In contrast, after removing the variance associated with affective-interpersonal traits, lifestyle-antisocial characteristics (marked by poor inhibitory control) were positively associated with responses in these regions, consistent with evidence showing that these traits are associated with hyperreactivity to emotional stimuli and poor emotional and behavioral regulation in both extreme and typical samples (Carré et al., 2013; Hyde et al., 2014; Patrick, Hicks, Krueger, & Lang, 2005; Seara-Cardoso et al., 2012). Our results are in line with and provide further evidence for the notion that, although the two dimensions of psychopathy co-occur, they may tap into two distinctive underlying constructs. These constructs share components, but also present unique aspects (i.e., those not shared with the other) that are related to distinct types of atypical emotional processing (Patrick, Hicks, Nichol, & Krueger, 2007). For example, variance in lifestyle-antisocial behaviors in the general population may stem from multiple sources. Individuals who present with these behaviors may do so because they lack empathy and concern for others (low emotional reactivity), or they may show reactive aggression to threat (increased emotional reactivity). Once the variance shared with affective-interpersonal traits is controlled for, what is left is variance in lifestyle-antisocial behavior that is driven by factors other than those that are held in common with affective-interpersonal traits. Likewise, individuals with high levels of affective-interpersonal traits may differ in their levels of antisocial behavior. Individuals with high levels of affective-interpersonal traits but low levels of lifestyle-antisocial behavior seem to present significantly higher education and intelligence than those with high levels of both affective-interpersonal traits and antisocial behavior (Mokros et al., 2015). These two groups have been referred to as “manipulative” and “aggressive” psychopaths respectively, illustrating their distinct behavioral profiles (Hervé, 2007; Mokros et al., 2015). The neurocognitive profiles of these two groups have not been explored, and it would be of interest to assess whether their distinct patterns of behavior rest upon distinct patterns of emotional reactivity.

It should be noted, however, that although the use of partial correlations is a powerful and informative technique to identify associations between different variables, it also poses some difficulties in the interpretation of results (Lynam, Hoyle, & Newman, 2006). The most important one is the difficulty of knowing exactly what construct is left once the variance of another correlated construct is removed (Lynam et al., 2006). A replication of the present findings using a larger sample with a group-comparison approach, with groups being defined by high and low levels on the two dimensions, would provide important further validation of these results. However, it is worth noting that this approach has its own limitations; for example, owing to the moderate positive correlation between the two dimensions, individuals high on one dimension but not the other may be difficult to recruit and somewhat unrepresentative of how these traits are distributed.

Finally, it is worth noting the pattern of associations found in midCC/ACC. We found significant associations between midCC response and both dimensions of psychopathic traits, whilst the association between ACC response was significant with lifestyle-antisocial traits and at trend with affective-interpersonal traits. According to the meta-analyses conducted by Fan et al. (2011) and Lamm et al. (2011), the region implicated in empathy for pain spans the border between these two regions. However, in spite of these associations with individual differences, we did not detect a main effect of pain > no-pain in these regions. Correlations and main effects are statistically distinct and, for any given region and any given process, each can be observed in isolation or both can occur (Calder, Ewbank, & Passamonti, 2011). When a robust correlation with a personality trait is found in the absence of a group main effect, this likely occurs because lower and higher scores on the personality dimension are associated with relative reductions and increases in the neural response. This produces an overall effect that does not significantly differ from zero, thus rendering the main effect of the task manipulation in that region nonsignificant.

### Conclusions

In summary, we have demonstrated that neural responses to others’ pain in the AI, IFG, and midCC, regions typically associated with empathic processing, are associated with variation in psychopathic traits in the general population. Strikingly, the two dimensions of psychopathy presented opposite associations with neural responses in these regions. These results provide further evidence for the notions that atypical function in these regions might represent neural markers of disrupted emotional and empathic processing for individuals with high levels of psychopathic traits; that the two dimensions of psychopathy tap into two separable constructs with distinct underlying vulnerabilities; and, finally, that the relationships observed at the extreme end of the psychopathy distribution apply to the nonclinical distribution of these traits in the general population—that there are continuities in the mechanisms that underlie psychopathy.

## Electronic supplementary material

Below is the link to the electronic supplementary material.

ESM 1 (PDF 28 kb)
