# Supplementary material for: Neural responses to others’ pain vary with psychopathic traits in healthy adult males
Source: Cogn Affect Behav Neurosci. 2015 Mar 17;15(3):578–88. doi: 10.3758/s13415-015-0346-7 (PMC4526584; doi:10.3758/s13415-015-0346-7)
Supplement: Supplementary file 1 — (PDF 28 kb) [file 13415_2015_346_MOESM1_ESM.pdf]

Table S1. Whole brain analyses showing main-effects across all participants for No-Pain> Pain BOLD response

	Peak						Cluster	
**Brain regions**	**L/R**	**x**	**y**	**z**	**t**	**Z**	**Extent (k)**	***P* (FWE)**
Calcarine sulcus	L	-8	-88	12	5.42	4.73	1416	<.001
White matter	R	28	-48	14	5.20	4.58	73	.39
Superior frontal gyrus	R	18	58	2	4.57	4.12	363	<.001
White matter	L	-30	-44	0	4.42	4.00	21	.92
Superior frontal gyrus	L	-14	50	-10	3.91	3.61	36	.77
Caudate nucleus	L	-18	24	4	3.96	3.65	12	.98
Superior frontal gyrus	R	24	-44	-34	3.96	3.65	15	.96
Supramarginal gyrus	R	44	-52	34	3.84	3.55	24	.90
White matter	L	-24	38	10	3.59	3.35	16	.96

Notes: Whole-brain analyses reported at a threshold level of $P < .001$ (uncorrected, cluster size > 10 voxels). Spatial coordinates (x, y, z) are in Montreal Neurological Institute space. R = Right; L = Left.

For Review Only
